# Walking and talking the tree of life: Why and how to teach about biodiversity

**DOI:** 10.1371/journal.pbio.2001630

**Published:** 2017-03-20

**Authors:** Cissy J. Ballen, Harry W. Greene

**Affiliations:** 1 Department of Ecology & Evolutionary Biology, Cornell University, Ithaca, New York, United States of America; 2 Department of Biology Teaching and Learning, University of Minnesota, Minneapolis, Minnesota, United States of America

## Abstract

Taxonomic details of diversity are an essential scaffolding for biology education, yet outdated methods for teaching the tree of life (TOL), as implied by textbook content and usage, are still commonly employed. Here, we show that the traditional approach only vaguely represents evolutionary relationships, fails to denote major events in the history of life, and relies heavily on memorizing near-meaningless taxonomic ranks. Conversely, a clade-based strategy—focused on common ancestry, monophyletic groups, and derived functional traits—is explicitly based on Darwin’s “descent with modification,” provides students with a rational system for organizing the details of biodiversity, and readily lends itself to active learning techniques. We advocate for a phylogenetic classification that mirrors the TOL, a pedagogical format of increasingly complex but always hierarchical presentations, and the adoption of active learning technologies and tactics.

## Introduction

The evolutionary tree of life (TOL) is the core of biology, a representation of the history of organismal diversification. Its more than 1.5 million species, with their characteristic, highly diverse genotypes and phenotypes, are the very phenomena that biology seeks to explain. Accordingly, we believe that all biology majors, to maximize their educational potential and regardless of career goals, should have a working knowledge of the TOL. As examples, every student with an undergraduate degree in biology should know the meaning of “bacteria,” “archaeans,” and “eukaryotes,” as well as that not all plants make flowers, and fungi are more closely related to animals than to plants. Likewise, every biology major should be able to specify, e.g., the diagnostic characteristics of mammals, that reptiles are their closest relatives, and that mammals plus reptiles collectively are known as amniotes. Furthermore, no 21st century physician asked by a patient, “well doc, what is *Giardia* anyway?” should answer that it’s a bug, a germ, a virus, or a bacterium—all of which are incorrect. Surely, a modern premedical student can have learned a handful of eukaryote clade names and then be able to explain that *Giardia* is a one-celled organism with a nucleus (unlike bacteria and viruses) in a group called excavates, which also includes the human pathogens *Trypanosoma brucei* (sleeping sickness) and *Trichomonas vaginalis* (trichomoniasis, a sexually transmitted disease). Furthermore, our students should be able to explain why drugs used for bacterial infections won’t work against *Giardia* and why those drugs that do work are more likely than antibiotics to also be toxic to human cells.

Traditionally, learning taxonomy has been an exercise in memorizing rank-based names, one that is supremely boring, provides limited and sometimes misleading information, and is increasingly irrelevant. Here we first highlight differences between traditional rank-based and clade-based approaches to teaching the TOL, then discuss the prevalence of each classification system among high school and university textbooks. We next describe how to teach biodiversity using clade-based taxonomy, an approach we call “walking and talking the tree of life.” Our method was developed over the past several decades while teaching herpetology at Berkeley and nonmajors introductory biology at Cornell (Greene) and an evolution and biodiversity course, required of all majors, at Cornell (Ballen and Greene). It has been modified repeatedly over the years in response to input from students, teaching assistants, and colleagues. Our clade-based method focuses on monophyletic groups and the often-adaptive homologies that underlie them, rather than simply the memorization of names and their ranks. Our overall purpose here is to lay out the pedagogical advantages of teaching the TOL from a phylogenetic perspective, with emphasis on active learning. We hope others will try it.

## Rank-based versus clade-based approaches to categorizing biodiversity

When Linnaeus updated his *Systema Naturae* in 1758, around which modern taxonomy was subsequently structured, there were 4,236 identified species of animals and 6,000 identified species of plants [1]. The traditional rank-based hierarchy places all of life into kingdoms, then into further “ranked” taxa as phyla, class, order, family, genus, and species. These ranked categories have always been potentially confusing (see below), and with more than 2 million species currently described and millions that have not yet been discovered [2, 3], accommodating the ever-branching TOL within that framework is increasingly ponderous—now ranks are further subdivided with prefixes, e.g., mag-, super-, grand-, mir-, sub-, and infra-.

Moreover, in light of ever more crowded species lists and the imminent expansion of taxa, what information does one gain from ranks? The common rejoinder that they provide information about numbers of species and/or relative diversification within groups is demonstrably untrue—traditionally ranked snake families include from one to hundreds of species [4], and more dramatically, order Coleoptera (beetles) contains >300,000 species, up to 20% of all described biodiversity [5], whereas order Primates, which includes humans and chimpanzees, encompasses ~400 species [6]. Likewise, in terms of morphological diversity among taxa within a group, phylum Arthropoda, which includes the hyper-speciose crustaceans and insects, is enormously more diverse than phylum Orthonectida, which includes only worm-like marine parasites [7]. Clearly we lack consistent criteria for grouping organisms in a ranked classification.

**Fig 1:**
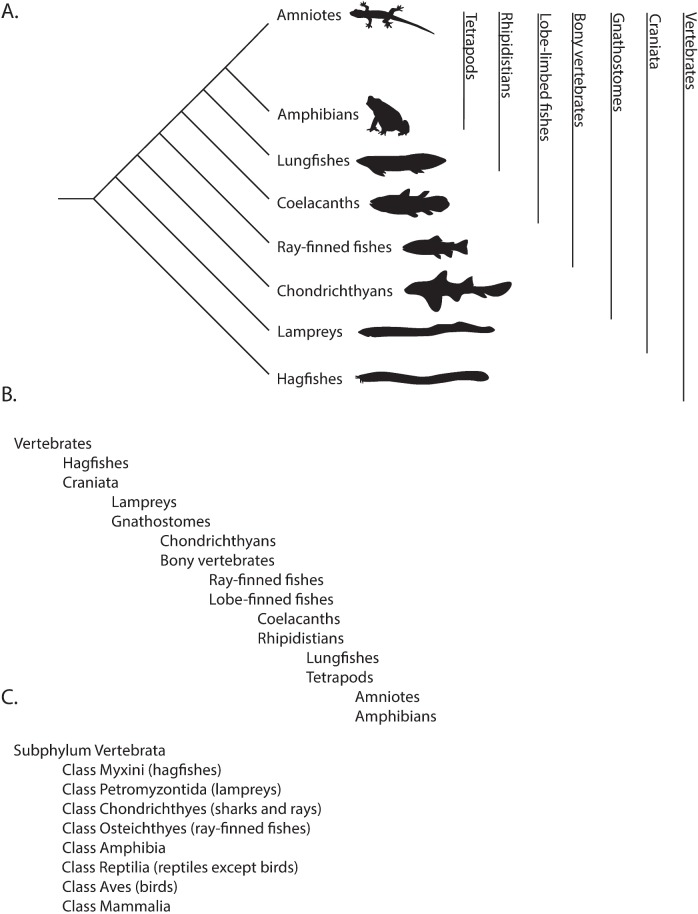
(A) A phylogenetic tree reflects the evolutionary relationships among major living vertebrate taxa. The vertical bars represent monophyletic groups that encompass corresponding taxa on tree tips. (B) The indented classification of major vertebrate taxa. Note how the indented classification correctly portrays the relationships depicted by the phylogenetic tree. (C) Traditional rank-based classification of the same living vertebrate taxa is inconsistent with portrayals in (A) and (B). The traditional classification obscures relationships among taxa, e.g., some Osteichthyes (coelacanths, lungfish) are more closely related to tetrapods than to other “fishes”; it also fails to denote three huge evolutionary innovations in the history of vertebrates: jaws, four limbs, and the amniotic egg.

More importantly, in terms of representing the history of life and as illustrated in Fig 1, rank-based classifications fail on two counts: they omit taxonomic recognition of major evolutionary innovations and inaccurately portray that history [8–11]. Traditional organization of vertebrates within eight class ranks, for example, omits specifying clades defined by the origin of limbs in tetrapods and of the shelled egg in amniotes. At the same time, those ranks completely obscure the fact that crocodilians are more closely related to birds than they are to turtles, lizards, and snakes, thus failing to highlight a number of dramatic similarities between the former groups of archosaurs (see below). And the traditional recognition of Osteichthyes as bony fishes, comprised of tens of thousands of species of ray-finned fishes in one lineage and a handful of coelacanths and lungfishes in the other, falsely implies an extremely unbalanced radiation of those two groups when, in fact, they are roughly equivalent in species numbers when the latter appropriately includes tetrapods.

Phylogenetic classification also reduces unnecessary memorization for students, who are still required to know the names of clades but not traditional rank names. Instead, phylogenetic taxonomy lends itself to a deeper understanding of biodiversity while placing it in larger evolutionary contexts. Students are encouraged to think about the relationships between taxa in terms of descent from common ancestors with fascinating clade-typical features, rather than associating taxa with corresponding ranks; as such, they learn taxa (names of nodes and branch tips in the TOL) through a successively more detailed but always completely hierarchical taxonomy in a fashion we will detail below.

While this change in how biodiversity is named is increasingly widely accepted among evolutionary biologists, the extent to which high schools and universities have adopted a phylogenetic approach to teaching has remained uncertain, granting significant “time lags” between research-inspired innovations and what we teach in classrooms [12]. Thus, in the next section, we discuss the discrepancy between primary literature and textbooks with respect to biological taxonomy.

### Textbooks present rank-based classification: Implications and solutions

**Fig 2:**
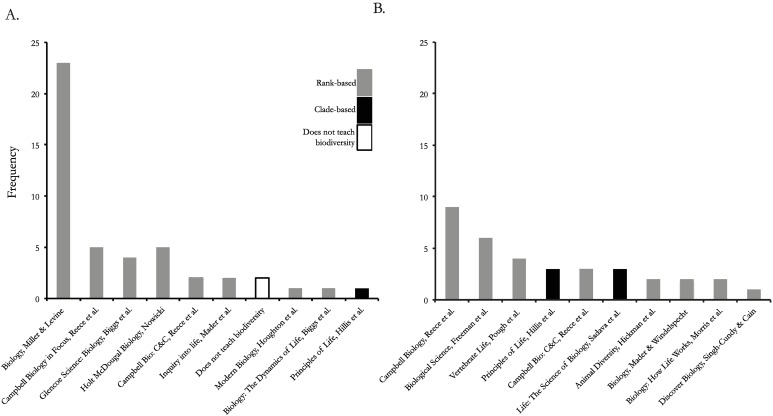
(A) The top ten most frequently used textbooks (*n* = 46 textbooks) by 46 US high schools, according to high school teachers. At present, many American high schools continue to use aspects of the traditional rank-based system (gray bars), as opposed to a clade-based classification system (black bars), to assign taxon names in the tree of life. White bars reflect high school texts that do not teach biodiversity. (B) The top ten most frequently used textbooks (*n* = 35 textbooks) by 50 large US universities to teach biodiversity.

Early on in the pursuit of a biology degree, students learn parts of the earth’s biological taxonomy. To infer the extent to which university and high school students are taught ranked-based taxonomy, we did the following: (1) Identified the single largest United States four-year university in each state using CollegeStats.org, which collates publicly available information from the US Department of Education. We then used the university’s website to find when biodiversity is first introduced to students on a biological sciences track and identified the corresponding textbook that accompanies the course (Fig 2A). We only included universities that had this information readily available on a class website. (2) Textbook information is not readily available for the high school level, so Ballen attended the National Association of Biology Teachers conference (November 2015) and asked 46 randomly chosen high school teachers which textbook they used (Fig 2B). With this information and the conservative assumption that all schools adopt the most recent textbook edition, we examined the text contents to see whether they use ranks. For our purposes, texts that identified supra-generic ranks are considered to use rank-based classification.

The majority of universities and high schools we surveyed use textbooks that teach some rank-based classification above the genus level. When we examined the top ten most frequently used textbooks, 30 out of 35 for universities (85%) and 45 out of 46 for high schools (98%) teach at least partly rank-based classifications, although many present partially clade-based phylogenetic taxa as well. For instance, many textbooks describe the origins of the traditional system and introduce its ranked terminology, while often making clear that the system has outlived its usefulness. This may be for the benefit of instructors who would be otherwise hesitant to replace the traditional ranking system. Despite this content warning, however, terms such as “phylum” and “kingdom” are subsequently used, along with outdated divisions among groups. For instance, some textbooks separate birds (“class Aves”) from reptiles (“class Reptilia”) and place crocodiles in among reptiles, despite the more than century-old consensus that birds and crocodiles are each other’s closest living relatives, such that birds are phylogenetically nested within reptiles. Even when the correct relationships are clarified in text, a traditional classification of birds as a separate class fails to reveal that derived traits of all living archosaurs (i.e., crocodilians and birds) include nest construction and parental care, a four-chambered heart, and acoustic communication. Thus, teaching phylogenetic taxonomy more accurately reflects evolutionary history and is more easily learned because it provides a clearer context to emphasize captivating homologous traits and descent with modification. Using textbooks that present the diversity of life in a rank-based classification teaches group names that may actively mislead students because ranks do not necessarily reflect evolutionary history, as in the case of birds and crocodilians. Other textbooks only briefly introduce rank-based classifications with the “Five Kingdoms” before describing the modern use of molecular tools and phylogenetic methods that have replaced them. While we also consider these textbooks to use “rank-based” classification, their blended approach is not as misleading if they stick with the principles of phylogenetic taxonomy for the remainder of the text.

There are several limitations to our findings. First, we assume high schools and universities are using the most recent edition of textbooks, but based on Ballen’s conversations with high school teachers, this was rarely the case. In fact, some teachers used textbooks over ten years old. Furthermore, we interviewed high school teachers while attending a teaching conference, and this sample may not be representative of high school teachers across the US. Many publicly funded high schools are not able to invest in this type of professional development. Finally, anecdotal statements from high school teachers suggest that in recent years, high schools and universities have placed less of an emphasis on teaching biodiversity in favor of molecular biology and genetics. Our conservative conclusion from this survey is that US high schools and universities are generally teaching biodiversity with an approach that is inaccurate and inefficient. We predict that the failure to use a truly evolutionary context for naming organisms on the TOL may be much more extensive than we can demonstrate at this point. These points also illustrate some of the obstacles institutions face at both the instructor- and logistical-level that may prevent them from teaching updated methods of classification.

## The TOL approach to teaching about biodiversity

Evolution and Biodiversity is a required introductory core course for all Cornell University biology majors. It typically enrolls ~250 students, mostly freshmen, every semester, is team-taught, and entails three 50-minute lectures and one 50-minute discussion section each week. Biodiversity amounts to one third of the course, or 12 lectures.

**Fig 3:**
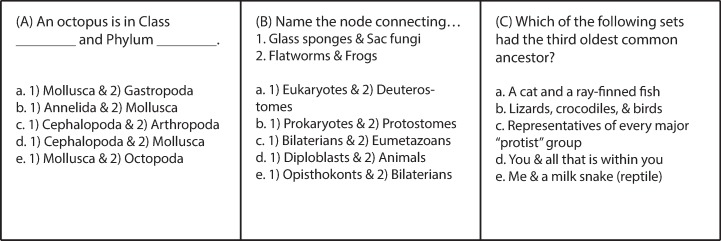
Three typical test questions reflect student expectations in introductory Evolution and Biodiversity. (A) Shows a typical question from a course that teaches rank-based classification, whereas (B) and (C) requires that students understand higher-order principles beyond memorization after learning a phylogenetic classification of diversity. (Answers: d., e., a.)

**Fig 4:**
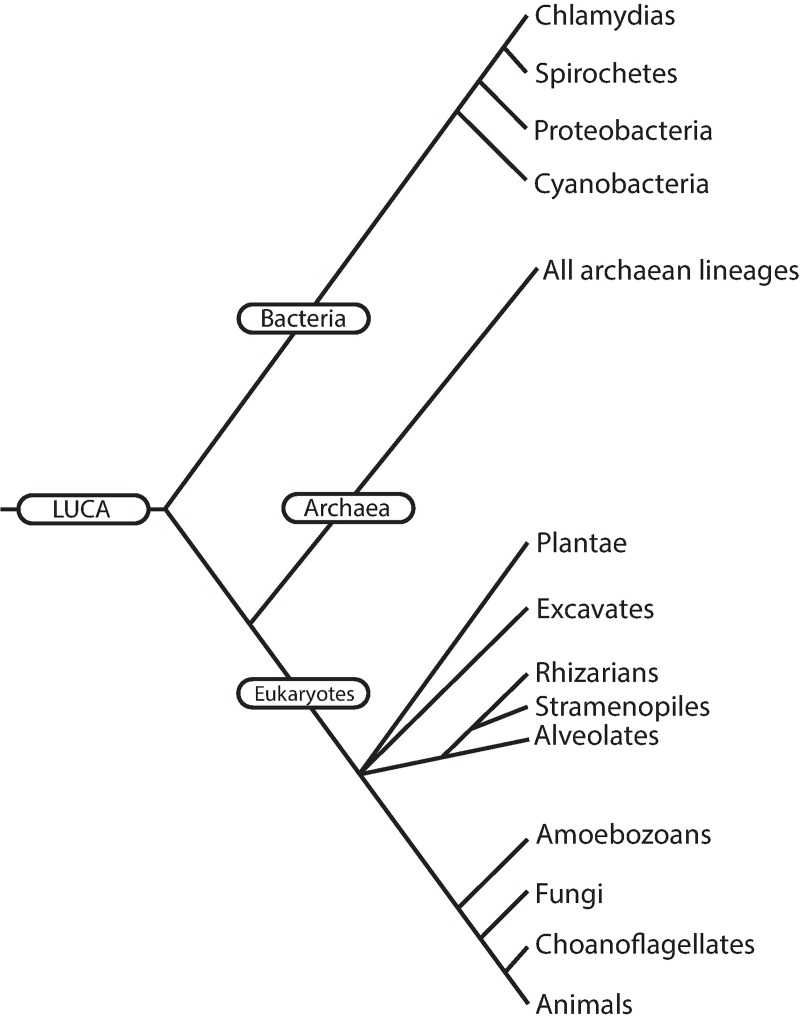
A “pruned” phylogenetic tree, starting at the Last Universal Common Ancestor (LUCA), reflects the evolutionary relationships among 17 major taxa and serves as a starting point for learning the tree of life in an introductory biology course at Cornell University (generated from [14]).

When we begin the module on biodiversity, we present the TOL as hierarchical, a system that can be expanded or collapsed depending on the level of taxonomic detail to be covered. Our introductory lecture is crucial in laying out this rationale, and we first present our total of ~145 taxa scrambled on three panels, noting that students will undoubtedly recognize the names of some well-known taxa (e.g., vertebrates, mammals, fungi), human pathogens (e.g., *Plasmodium*, *Chlamydia*), and model organisms for research (e.g., *Saccharomyces cerevisiae*, *Caenorhabditis elegans*). Then we explain that, rather than using Linnaeus' “*Systema Naturae*” or traditional rank-based, we will navigate an indented phylogenetic classification that strictly replicates the topology of the evolutionary historical TOL (Fig 1). We assure students that if they buy into our approach, by the end of the 12 lectures, we can place them at any branch tip or node, specify another location on the TOL, and they will be able to virtually navigate between the two points. Students list the nodes they pass through from their starting taxon until they reach the most recent common ancestor that it shares with the second taxon; then they must list all the nodes as they move back “up” the tree towards the tips until they arrive at the second taxon. In Fig 3C, students must virtually navigate the TOL in order to identify which group of taxa, of multiple choices, shares the third most recent common ancestor in the TOL. As we repeatedly emphasize, all of this is elegantly nonrandom and represents Darwin’s “descent with modification.” It integrates molecules to organisms, structurally and functionally, across space and time. It has profound scientific, medical, and societal implications [13]. In the beginning, we present the simplest representation of life on earth, one with the basal node “LUCA” (representing the Last Unique Common Ancestor of life as we know it) and three terminal taxa—Bacteria, Archaea, and Eukaryota. Then, we add in a half dozen clades of unicellular eukaryotes to bring the total to almost a dozen, and so on (Fig 4). Throughout the module we repeatedly expand and collapse the TOL to remind students where we are at, where we have been, and where we are going. We throw in examples that may surprise them or reinforce the patterns they’re learning: that sea stars and urchins are more closely related to us than to other “invertebrates;” that powered flight evolved independently in insects, pterosaurs, birds, and bats; and that multicellularity evolved repeatedly, even, arguably, in some bacteria.

## Active learning and some general principles to teaching the TOL

In fall 2015, Evolution and Biodiversity transitioned from a traditional passive lecture format to an active-learning curriculum, with focus on student-centered pedagogies such as collaborative in-class group work and discussion to increase student engagement. The incorporation of active learning led to challenges and opportunities in our approaches to teaching biodiversity. In fall 2015, we implemented the following changes to the course: (1) students were randomly assigned to groups of five, with whom they sat for the entire semester; (2) students were required to take low-risk assessments before every class period in the form of prelecture quizzes that tested their knowledge of materials from prelecture assignments, including video podcasts (“vodcasts”) and textbook readings; (3) students used iclickers, a personal electronic response system, to answer multiple-choice questions throughout class, or their group number was called on to contribute with the use of a random-number generator and a catchbox microphone; (4) students were required to participate in class discussions and active-learning exercises. These changes to the course dramatically altered how we presented content in lecture. For example, before attending a lecture about Fungi, students watched two vodcasts that served as a brief introduction to the major clades within Fungi and the characteristics members of each clade share (these characteristics are called “synapomorphies”). Thus, class time was spent reinforcing this material instead of introducing it. In the lecture about Fungi, we introduce a “Fungal Mycology Mystery” activity. It requires that students use backwards elimination to identify the clade from which a sample belongs.

Several key principles underlie our approach to teaching biodiversity. First, we strive to use monophyletic taxa whenever possible, i.e., a common ancestor and all of its descendants. We highlight several traditional names that we predict will soon become extinct—prokaryotes, protistans, invertebrates, algae—as well as others that are rapidly being accepted in redefined monophyletic fashion, like reptiles (including birds). Second, we prune the tree down to a reasonable number, considering the time allotted to teaching and completing course learning goals. Over the course of 12 lectures, for each major clade, we provide lists of required taxa and terms as well as a road map to broader contexts for appreciating each group. For alveolates, we briefly cover dinoflagellates and ciliates, with some emphasis on *Paramecium*, as an example of detailed structure in a unicellular organism, and on *Plasmodium*, to illustrate complex life cycles in a medically important parasite. We emphasize nodes characterized by relevant adaptations and interesting macroevolutonary implications: the invention of the amniotic egg gains special significance once students view the terms amnion and chorion in a figure of human placental structure and function—and all the more so once we explain that among all amniotes, live bearing has arisen once within mammals, never among more than 10,000 species of birds, crocodilians, and turtles, and yet it has evolved more than 100 times among extant lizards (including snakes). For the benefit of premed students, we code branch tip taxa that include at least one human pathogen with a skull and crossbones symbol. In the final lecture, we place our ability to walk and talk the TOL in the general context of other courses in biology, as well as appreciating biodiversity as citizens—a student from the nonmajors course once approached Greene on campus and related that he’d just seen a pine tree and said to himself, “Now that’s not an angiosperm!”

**Fig 5:**
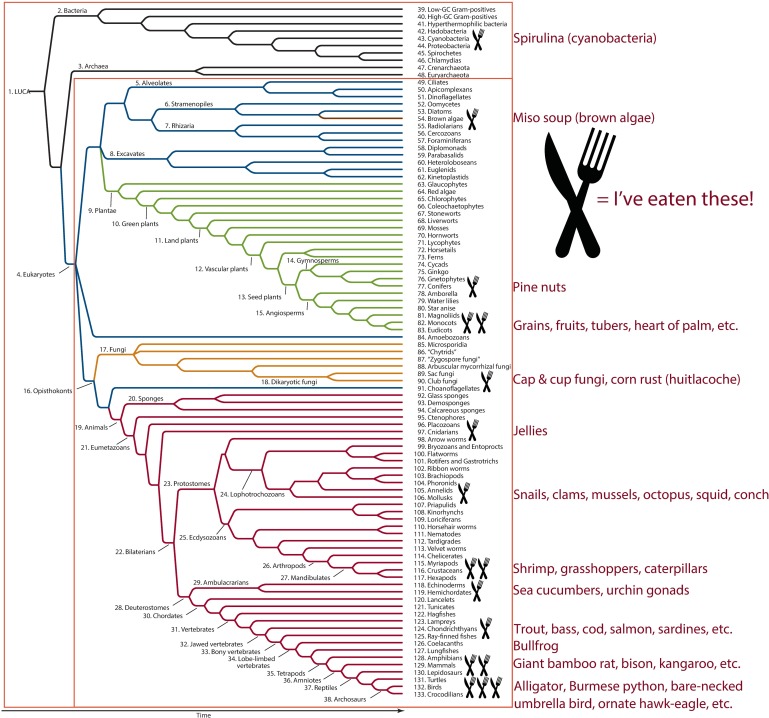
To summarize the tree of life module in our introductory evolution and biodiversity course, Greene expands his Lifetime Phylogenetic Dietary Diversity from Eukaryotes (inner red box) to LUCA (outer red box) by consuming a *Spirulina* capsule in front of the class (phylogeny used with permission from Sinauer Associates, Hillis et al. 2014).

From a pedagogical perspective, teaching descent with modification rather than emphasizing named ranks facilitates the development of more challenging in-class activities and exam questions. Fig 4 shows an example of three test questions attempting to assess students’ ability to determine nested monophyletic groups of organisms. The first question tests whether students can identify the taxa assigned to ranks “class” and “phylum” for an octopus by filling in the blank. The second and third questions test comprehensive knowledge and require higher-level critical thinking skills to answer. Students have to think about where organisms are located on the TOL and trace the history of two taxa back to a common ancestor (node). And we watch for ways to actively engage them even during lecture components of the course. We scored with a fork and spoon symbol, for example, the particular branch tip taxa the instructor has consumed and in the final offering, with our entire TOL in view, asked for the smallest inclusive taxon for his Lifetime Phylogenetic Dietary Diversity (LPDD)—immediately yielding a resounding “Eukaryotes!” from the class (Fig 5). He next proposed right then to expand his LPDD in front of them, pulled out a water bottle and swallowed a huge blue-green *Spirulina* capsule, added a fork and spoon symbol to cyanobacteria on the TOL and asked, “now what is the smallest taxon for all the marked branch tips?”—at which point the class thundered, “LUCA!” (the Last Universal Common Ancestor) and broke into applause.

It can be challenging for students to visualize where taxa are located on the TOL, but there are an increasing number of online resources available for use as study tools. The TimeTree iPhone/iPad application generates the amount of time since two taxa split from their most recent shared ancestor and lets you visualize the split on a TOL. The Tree of Life iPhone/iPad application tests the identification skills of users by providing spaces to fill in the blank on phylogeny nodes and tips, given a limited amount of information on their position relative to other taxa. Lifemap allows students to navigate between branch tips and nodes of phylogenetic trees using a zoomable interface [15]. Textbooks are increasingly adding online interactive components specifically tailored to the diversity portions of texts, and there are websites available that further test students’ knowledge (e.g., University of Michigan Museum of Zoology Animal Diversity Web, The Howard Hughes Medical Institute website, the Berkeley evolution website, the Tree of Life website, and Open Tree of Life websites). In our evolution and biodiversity core course, students are required to purchase an evolution text and a biodiversity reference. Currently, we find that the five diversity chapters in Hillis et al. 2014 [14] nicely fit our goals in that somewhat more taxa are covered than we require, the approach is strictly phylogenetic and nonranked, and the book’s publisher provides a bundled version of those chapters plus the overall text’s glossary. We tell students that this is the most important text they will purchase as a biology major, the one they shouldn’t sell and to which they will refer throughout the rest of their undergraduate years.

The evolutionary TOL is the scaffolding on which the rest of biological study relies. At present, the majority of science students learn core taxa through memorization, associating groups with arbitrary ranks instead of emphasizing that they are descendants from a common ancestor. However, change is slowly occurring. The promotion of a phylogenetic approach to learning the TOL will be made possible through continued dissemination of relevant research and systemic change among our high schools and universities.

## Abbreviations

LPDD: Lifetime Phylogenetic Dietary Diversity
LUCA: Last Universal Common Ancestor
TOL: tree of life
